# Efficacy and safety of recombinant activated factor VII for secondary prophylaxis in patients with Glanzmann thrombasthenia

**DOI:** 10.1016/j.rpth.2026.106792

**Published:** 2026-06-13

**Authors:** Pablo García-Jaén, Nuria Fernández-Mosteirin, María Luisa Serrano, Susana Riesco, Carlos Aguilar, David Gabaldon, Sara Kanaan-Leis, Almudena González-Prieto, Raquel Portugal, María Carmen Mendoza, Carmen Gilabert, Ana Hortal, Lorena Díaz-Ajenjo, Ana Lama-Villanueva, Alba Mei González-Galán, Ana Sánchez-Fuentes, Rocío Benito, José Rivera, José Ramón González-Porras, José María Bastida

**Affiliations:** 1 Servicio de Hematología, Complejo Asistencial Universitario de Salamanca (CAUSA), Instituto de Investigación Biomédica de Salamanca (IBSAL), Universidad de Salamanca (USAL), Salamanca, Spain; 2 Servicio de Hematología, Hospital Universitario Miguel Servet, Zaragoza, Spain; 3 Servicio de Pediatría, Complejo Asistencial de Soria, Soria, Spain; 4 Servicio de Pediatría, Complejo Asistencial Universitario de Salamanca, Salamanca, Spain; 5 Servicio de Hematología, Complejo Asistencial de Soria, Soria, Spain; 6 Servicio de Pediatría, Complejo Asistencial Universitario de Burgos, Burgos, Spain; 7 Centro de Investigación del Cáncer—CSIC—Universidad de Salamanca (USAL), Instituto de Investigación Biomédica de Salamanca (IBSAL), Salamanca, Spain; 8 Servicio de Hematología, Hospital Universitario Morales Meseguer, Centro Regional de Hemodonación, Universidad de Murcia, IMIB-Pascual Parrilla, CIBERER-U765, Murcia, Spain; 9 Coordinadores del Grupo Español de Alteraciones Plaquetarias Congénitas (GEAPC), Spain

**Keywords:** bleeding, Glanzmann thrombasthenia, prophylaxis, recombinant activated factor VII

## Abstract

**Background:**

Glanzmann thrombasthenia (GT) is a rare inherited platelet disorder associated with recurrent and sometimes life-threatening bleeding. Recombinant activated factor VII (rFVIIa) is approved for treatment of bleeding episodes or surgical prophylaxis in patients with GT who are refractory or alloimmunized to platelet transfusions. However, evidence for its use as secondary prophylaxis remains scarce.

**Objectives:**

This study described the efficacy and safety of rFVIIa secondary prophylaxis in patients with GT and recurrent and uncontrolled bleeding despite standard therapy.

**Methods:**

A multicenter, retrospective study of patients with GT included in the Spanish Registry of Patients with Inherited Platelet Disorders (RETPLAC) was performed. We evaluated bleeding severity using the International Society on Thrombosis and Haemostasis (ISTH) bleeding assessment tool (BAT) and calculated the annualized bleeding rate (ABR) before and during rFVIIa prophylaxis.

**Results:**

Of the 37 patients with GT included in RETPLAC, 4 patients received regular rFVIIa prophylaxis due to recurrent severe bleeding. Median baseline ISTH-BAT score was 13.5 (IQR, 9.5-18), and median preprophylaxis ABR was 3 (IQR, 3-5.25). All patients received rFVIIa of 90 μg/kg, 3 twice weekly and 1 monthly. The median prophylaxis duration was 7.5 months (IQR, 6.75-12). Prophylaxis led to a 57.1% overall reduction in ABR, with resolution of severe bleeding in 2 patients and reduction in frequency in the remaining 2. Three patients achieved transfusion independence. All patients showed improvement in hemoglobin and iron profile. No thrombotic or adverse events occurred.

**Conclusion:**

Secondary prophylaxis with rFVIIa may be an effective option in patients with GT and recurrent severe bleeding unresponsive to standard therapy. Prospective studies are necessary to optimize dosing schedules and define its role in secondary prophylaxis.

## Essentials

• Secondary prophylaxis options for patients with Glanzmann thrombasthenia remain limited.
• Evidence supporting recombinant activated factor VII (rFVIIa) for secondary prophylaxis is scarce.
• Four patients from the Spanish registry received scheduled rFVIIa for recurrent severe bleeding.
• Secondary prophylaxis reduced bleeding, avoided transfusions, and showed no safety concerns.

## 1 Introduction

Glanzmann thrombasthenia (GT) is a rare autosomal recessive bleeding disorder caused by quantitative or qualitative defects in the platelet glycoprotein (GP) IIb/IIIa (α_IIb_β_3_ integrin) complex, resulting in impaired platelet aggregation [1,2]. Management of GT remains challenging, as patients frequently experience recurrent mucocutaneous bleeding, which can become life threatening [[1], [2], [3], [4]]. Platelet transfusion is the standard treatment for preventing and controlling severe bleeding, but its effectiveness is often compromised by platelet refractoriness [5]. Patients with GT, particularly when GPIIb-IIIa is absent, may develop isoantibodies against α_IIb_β_3_. Repeated platelet transfusions may also lead to immunization, including anti-human leukocyte antigen alloantibodies, which may contribute to platelet refractoriness. Some patients continue to experience recurrent bleeding despite the treatment. Beyond the physical burden, recurrent bleeding episodes significantly impact patients’ psychological well-being and overall quality of life, particularly among women with heavy menstrual bleeding (HMB), as highlighted by Glanzmann’s 360 Study Working Group [6]. These findings emphasize the importance of effective hemorrhagic prophylaxis strategies.

Recombinant activated factor VII (rFVIIa) has proven effective in controlling bleeding and providing perioperative prophylaxis in patients with GT who are refractory to or alloimmunized against platelets [[7], [8], [9], [10], [11]]. For this reason, the European Medicines Agency approved rFVIIa for use in patients with GT who were refractory to platelets or when platelets were not readily available [12]. Some authors have suggested that early administration of rFVIIa during bleeding may reduce the risk of alloimmunization by limiting the exposure to platelet transfusions, thereby reserving them for more critical situations [9]. However, its role for long-term secondary prophylaxis of recurrent bleeding remains unclear, with current evidence limited to isolated case reports and small case series [[13], [14], [15], [16]]. In this study, we evaluated the efficacy and safety of rFVIIa for long-term secondary prophylaxis in patients with GT.

## 2 Methods

We conducted a retrospective, multicenter study focusing on patients with GT who required secondary prophylaxis with rFVIIa due to recurrent and/or severe bleeding episodes that did not respond to standard therapy, such as platelet transfusions, antifibrinolytics, and iron supplementation. To identify these patients, we analyzed data from the Spanish Registry of Patients with Inherited Platelet Disorders (RETPLAC; https://seth.es/investigacion/geapc/registro-espanol-de-trastornos-plaquetarios-congenitos-retplac/). This registry is managed by the Spanish Group of Congenital Platelet Disorders (GEAPC), overseen by the Spanish Society of Thrombosis and Hemostasis (SETH) and included in the European Directory of Registries (https://eu-rd-platform.jrc.ec.europa.eu/erdridor/register/name/RETPLAC; accessed February 13, 2026). The genetic study was performed using the targeted sequencing panel designed and reported by our group [17].

Of the 37 patients with GT included in RETPLAC, 4 were eligible for this study. The patients selected for hemostatic prophylaxis were identified according to the individualized clinical judgment of the treating physicians at each participating center. Bleeding severity was assessed using the International Society on Thrombosis and Haemostasis (ISTH) bleeding assessment tool (BAT) [18]. The annualized bleeding rate (ABR) was calculated for each patient during the 12 months prior and throughout the rFVIIa prophylaxis period. To evaluate the efficacy of the prophylaxis, the relative risk reduction in ABR was calculated. Hemoglobin values and the iron profile were measured at baseline and 4 to 6 weeks after the prophylaxis with rFVIIa was initiated. The duration and frequency of prophylaxis were tailored to each patient based on their clinical response. Transfusion independence was defined as the absence of any requirement for blood component transfusions from the initiation of secondary prophylaxis onward. Major bleeding events were classified according to criteria established by ISTH [19]. Adverse events were classified according to the Common Terminology Criteria for Adverse Events, version 5.0 [20]. Clinical and laboratory data were obtained from the RETPLAC and supplemented with additional information collected from participating centers. All data collection was conducted in accordance with institutional ethics committee approvals, and informed consent was obtained from all participants or their legal guardians. Due to the small sample size and the nonparametric distribution of the data, quantitative variables are reported as the median and IQR (Q1-Q3), while categorical variables are presented as absolute numbers and percentages. Statistical analyses were descriptive in nature, and no formal statistical hypothesis testing was performed. All analyses were conducted using IBM SPSS Statistics v28.0.1.1 [14].

## 3 Results and Discussion

Among the 37 patients included in the registry, the median age at diagnosis was 12 years (IQR, 2-32), and the median ISTH-BAT score was 13 (IQR, 8-18). Overall, 70% of patients were females, and 70% had type I GT. Among female patients, 65% had at least 1 gynecologic bleeding manifestation, while 27% fulfilled criteria for severe HMB (requiring hospital admission, blood transfusion, replacement therapy, and/or surgical procedure).

The study involved 4 patients (1 male and 3 females), with median age at the start of prophylaxis of 40 years (IQR, 29.8-47). The median ISTH-BAT score at GT diagnosis was 13.5 (IQR, 9.5-18). The clinical indications for prophylaxis included severe recurrent epistaxis with hypovolemic shock (patient [P]1), persistent gingival bleeding (P2), combined gingival and nasal bleeding causing anemia (P3), and HMB (P4). One patient (P2) met the criteria for platelet refractoriness (Table). The median preprophylaxis ABR was 3 (IQR, 3-5.25). To avoid overestimation, HMB was excluded from the ABR for P2 and P3 due to its predictable nature, but included for P4 as it was her sole bleeding manifestation and the indication for prophylaxis. Baseline full blood counts and iron profile are shown in the Table. The genetic study was performed in 3 patients (P1, P2 and P4) using a targeted sequencing panel designed and previously reported by our group [17]. It revealed pathogenic variants in *ITGA2B* and *ITGB3* genes (Table). At the time of reporting, genetic testing remained pending in 1 patient (P3). They were all diagnosed with type I GT. All patients received rFVIIa at a dose of 90 μg/kg. Three patients (P1, P2, and P3) were treated twice weekly, while 1 patient (P4) received monthly prophylaxis in line with her menstrual cycle. The median duration of prophylaxis was 7.5 months (IQR, 6.75-12). During prophylaxis, a 57.1% reduction in ABR was observed in terms of relative risk reduction. Two patients (P2 and P3) achieved complete cessation of severe bleeding, while P1 and P4 experienced a significant reduction in the frequency and severity of bleeding episodes (Table). Three patients (75%) became transfusion independent. No thrombotic or infusion-related adverse events were reported. In 1 patient (P3), the rFVIIa dosage was reduced from 90 μg/kg twice weekly to once weekly within the first 3 months of prophylaxis. Notable and generalized improvements were observed in hemoglobin and iron profiles, with median increases in hemoglobin of 1.45 g/dL, ferritin of 15 ng/mL, and serum iron of 15 μg/dL. It should be noted that 75% of patients were able to discontinue antifibrinolytic treatment once prophylaxis was established. Half of these patients also managed to reduce the dose or frequency of iron administration (Table). P2 and P3 remained on prophylaxis at the time of reporting. In P1, prophylaxis was stopped at the request of the patient and family, while in P4, it was discontinued by mutual agreement after all bleeding events disappeared.

**Table:** Clinical characteristics and prophylactic regimen in 4 patients with Glanzmann thrombasthenia treated with rFVIIa.

Variable	P1	P2	P3	P4
Current age (y)	13	47	56	36
Gender	Male	Female	Female	Female
Bleeding score	8	17	21	10
Bleeding phenotype	Epistaxis, bruising, gingival bleeding	HMB, mucocutaneous, GI bleeding	HMB, gingival bleeding	HMB
Glanzmann thrombasthenia	Type I	Type I	Type I	Type I
Gene	*ITGA2B*	*ITGB3*	*—*	*ITGA2B*
cDNA mutation	c.2748_2757del	c.431T>G	—	c.2944G>A, c.1612G>T
Protein change	p.Thr917Serfs∗181	p.Met144Arg	—	p.Val982Met, p.Glu538∗
Platelet refractoriness	No	Yes	No	No
Baseline				
Hb (g/dL)	9.7	10.2	11.1	10.1
MCV (fL)	78	102.6	87.1	89.5
Iron (μg/dL)	28	50	44	34
Ferritin (ng/mL)	42	705	99	36
Severe bleeding prior to prophylaxis	Yes	Yes	Yes	No
ABR before prophylaxis	12	3	3	3
Reason for starting prophylaxis	Recurrent severe epistaxis with hemorrhagic shock	Recurrent severe gingival bleeding with anemia	Gingival bleeding and recurrent epistaxis with anemia	Heavy menstrual bleeding
Hormonal contraception	—	Yes	No	Yes
Age at prophylaxis initiation (y)	11	44	56	36
Permanent venous catheter	Yes	No	No	No
rFVIIa dose (μg/kg)	90	90	90	90
Administration frequency	Twice weekly	Twice weekly	Twice weekly	Once monthly
Use of antifibrinolytics at starting of prophylaxis	Yes	Yes, oral TA, 500-1000 mg every 8 h if bleeding symptoms	Yes, oral TA, 500-1000 mg every 8 h if bleeding symptoms	Yes, oral TA, 500-1000 mg every 6-8 h when menstrual bleeding
Discontinuation of antifibrinolytics on prophylaxis	No	Yes	Yes	Yes
Iron supplementation at starting of prophylaxis	Oral (256 mg), once daily	Intravenous (200 mg), once weekly	Intravenous (200 mg), once weekly	Intravenous (200 mg), once monthly
Discontinuation of iron therapy on prophylaxis	No	Dose reduction to 200 mg every 2-3 wk	Dose reduction to 200 mg every 2-3 wk	No
Prophylaxis				
Hb (g/dL)	10.9	11.7	12.5	12.3
MCV (fL)	76	102.5	88.2	87
Iron (μg/dL)	25	71	55	53
Ferritin (ng/mL)	27	442	144	136
Local hemostatic treatment needed	NS	Topical adrenaline to control gingival bleeding	No	No
ABR during prophylaxis	3	3	3	0
Severe bleeding after prophylaxis	Yes	No	No	No
Need for rescue treatment	Yes (rFVIIa)	No	No	No
Duration of prophylaxis (mo)	8	24	6	7
Ongoing prophylaxis	No	Yes	Yes	No
Thrombotic complications	No	No	No	No

In patients with <1 year of prophylaxis follow-up, an annual estimate was established based on bleeding episodes during the follow-up period.

ABR, annualized bleeding rate; GI, gastrointestinal; Hb, hemoglobin; HMB, heavy menstrual bleeding; MCV, mean corpuscular volume; NS, not specified; rFVIIa, recombinant activated factor VII; TA, tranexamic acid.

This multicenter retrospective study provides preliminary evidence supporting the use of rFVIIa for secondary prophylaxis in patients with GT. Our findings showed that scheduled rFVIIa administration was associated with a clinically significant reduction in both the frequency and severity of bleeding, consistent with previous reports (Table) [[13], [14], [15], [16]]. The use of rFVIIa has emerged as an effective and safe therapeutic strategy for patients with GT, particularly those with anti-GPIIb/IIIa alloantibodies or who are refractory to platelet transfusions. An international multicenter survey led by Poon et al. [7] documented the efficacy of rFVIIa in both bleeding episodes and perioperative prophylaxis, reporting success rates >80% in refractory patients. Subsequently, Poon [8] expanded the clinical evidence, emphasizing rFVIIa as a first-line option when platelet transfusions are contraindicated or ineffective and highlighting its favorable safety profile. Recently, a systematic review consolidated these findings, by analyzing multiple studies and case reports supporting the hemostatic efficacy of rFVIIa in GT without a significant increase in thrombotic events [9]. These studies have demonstrated the pivotal role of rFVIIa in the management of refractory GT, supporting its incorporation into international clinical guidelines. However, modern studies have identified genetic variants that may be linked to reduced response to rFVIIa [21].

Until the development of novel agents are currently under investigation, effective prophylaxis against bleeding in patients with GT has remained an important unmet clinical need. In this context, case reports have introduced the use of rFVIIa as a novel approach for secondary prophylaxis in patients with GT experiencing recurrent bleeding episodes [[13], [14], [15], [16]]. Andiç et al. [13] administered a weekly low-dose regimen of rFVIIa to a patient with GT and platelet refractoriness. This resulted in a marked reduction in bleeding frequency and improved quality of life. Similarly, Laguna [15] and Lee et al. [14] described the successful use of rFVIIa to prevent intra-articular and gingival bleeding, as well as hemarthrosis in 3 patients. Recently, 2 case reports by Almatar et al. [16] exhibited excellent long-term responses to rFVIIa prophylaxis.

Our 4 cases provide additional evidence to support the use of rFVIIa as secondary prophylaxis in GT, demonstrating its effectiveness in treating a wider range of patients with different bleeding phenotypes. The individualized dosing schedules—including twice-weekly and cyclical monthly regimens—highlight the flexibility of rFVIIa in adapting to the specific needs of each patient. Notably, the successful use of monthly prophylaxis aligned with the menstrual cycle in a patient with HMB represents a novel and practical approach that has not been previously described. Overall, these findings reinforce the role of rFVIIa as a safe and personalized prophylactic strategy for patients with GT and severe or refractory bleeding.

Prophylactic rFVIIa could help to reduce transfusion requirements and prevent the formation of alloantibodies, while improving daily functioning and quality of life for affected families. Furthermore, improvement in biological parameters and/or the possibility of discontinuing or reducing the frequency of concomitant treatments are also associated with significant improvements in quality of life. Similarly, women of reproductive age often experienced HMB and are at risk of severe obstetric hemorrhage and neonatal alloimmune thrombocytopenia due to anti-α_IIb_β_3_ antibodies [5,8]. Therefore, limiting platelet exposure through prophylactic rFVIIa could provide additional benefits in controlling bleeding and mitigating long-term immunologic and reproductive complications [22]. Furthermore, this preventive strategy could contribute to improved psychosocial well-being. This is supported by Glanzmann’s 360 study, which reported high levels of distress related to bleeding and a reduced quality of life among women [6]. The apparent difference between our preprophylaxis bleeding rates and the high bleeding frequency reported in Glanzmann’s 360 study likely reflects differences in methodology and study objectives rather than conflicting clinical observations. The Glanzmann’s 360 study was a patient-reported study designed to capture the overall burden of disease in a broad GT population, including frequent minor mucocutaneous bleeding and its psychosocial consequences. In our study, the ABR was used as a descriptive measure of clinically relevant bleeding burden over a defined observation period, focusing on recurrent bleeding episodes in 4 highly selected patients with severe, difficult-to-control bleeding who required secondary prophylaxis. Accordingly, these datasets are complementary rather than directly comparable.

A recent Indian study provides an important global perspective on this issue. In that cohort, which included 64 patients with GT, the mean ABR was 11.85. However, none of the patients had received rFVIIa, and bleeding-related deaths were mainly attributed to the nonavailability or nonaccessibility of treatment products [23]. These findings suggest that reported bleeding rates should be interpreted in light of study design, patient selection, and access to specialized hemostatic care and reinforce the need to ensure that advances in GT management also reach patients in low-resource settings.

These previously unreported cases further expand the cohort of patients documented within the GEAPC framework [17]. Our study contributes to the limited existing body of evidence and represents the first multicenter study conducted in Spain. Despite these findings, several limitations must be acknowledged. The small sample size and retrospective design prevent definitive conclusions regarding efficacy and optimal treatment protocols from being drawn. It is important to take into account that patient selection was not based on a standardized protocol or on a predefined prospective selection strategy using only the clinical variables captured in the registry database. Rather, prophylaxis was prescribed as part of routine clinical care, reflecting individual assessment of bleeding risk and clinical need. Furthermore, variability in dosing frequency and duration reflects the lack of consensus on standardized regimens. These observations highlight the urgent need for prospective, multicenter studies to establish evidence-based guidelines for secondary prophylaxis in GT.

It is also worth noting the emergence of novel investigational prophylactic therapies, such as sutacimig, a subcutaneously administered bispecific antibody designed to localize endogenous FVIIa to activated platelets. Interim data from a phase 2 clinical trial (NCT06211634) have shown favorable results in patients with GT [24,25]. In addition, recent *in vitro* studies exploring the tissue factor pathway inhibitor concizumab have been published [26]. However, until these agents receive regulatory approval and become accessible outside clinical trials, their role in routine clinical practice remains uncertain. In this context, when participation in a clinical trial is not available, prophylactic use of rFVIIa may be considered a practical option for selected patients with severe, recurrent bleeding.

In conclusion, regular prophylaxis with rFVIIa may represent a safe and effective strategy to reduce bleeding in patients with GT who experience recurrent and/or severe bleeding unresponsive to standard therapy. In this small and heterogeneous series, rFVIIa prophylaxis was associated with a reduction in bleeding burden, although the magnitude of benefit was not uniform across all patients. The observed reduction in ABR and the achievement of transfusion independence suggest a sustained preventive effect on bleeding episodes and the overall disease burden. Beyond the physical impact, prevention of bleeding episodes may translate into meaningful improvements in patients’ quality of life. This study supports the feasibility of secondary prophylaxis and underscores the need for collaborative, prospective trials to establish evidence-based management strategies for this rare disorder.
